# The Current Knowledge, Attitudes, and Practices of the Neglected Methodology of Web-Based Questionnaires Among Chinese Health Workers: Web-Based Questionnaire Study

**DOI:** 10.2196/41591

**Published:** 2023-01-27

**Authors:** Heping Fang, Yuxin Lv, Lin Chen, Xuan Zhang, Yan Hu

**Affiliations:** 1 Department of Respiratory Medicine, Children's Hospital of Chongqing Medical University National Clinical Research Center for Child Health and Disorders Chongqing China; 2 Department of Child Health Care, Children's Hospital of Chongqing Medical University Chongqing Key Laboratory of Pediatrics, Ministry of Education Key Laboratory of Child Development and Disorders National Clinical Research Center for Child Health and Disorders Chongqing China

**Keywords:** epidemiological survey, knowledge, attitudes, and practices (KAP), medical education, methodology, web-based questionnaire (WBQ)

## Abstract

**Background:**

Web-based questionnaire (WBQ) surveys are popular, but the quality of reporting WBQ survey research is uneven and unsatisfactory worldwide. Education and training on WBQ methodology may be necessary. However, the current knowledge, attitudes, and practices (KAP) of its methodology remain unknown.

**Objective:**

We investigated the KAP of WBQ methodology among Chinese health workers for the first time to clarify the possible reasons for the unsatisfactory reporting quality of WBQ survey research from China’s experience, aiming to provide a basis for improvement.

**Methods:**

We developed a structured WBQ based on the current recommendations and knowledge and investigated 458 health workers from June 7 to July 6, 2022. A total of 381 valid questionnaires were analyzed after data processing. We defined 50% and 75% as “qualified” and “satisfactory” in knowledge and practice topics to describe the results and analyzed the basic characteristics of the participants who had difficulties in conducting WBQ survey research.

**Results:**

A total of 215 (56.4%) participants had used WBQs for investigation, mostly more than 2 times (88.3%), but only 95 (44.2%) of them had ever received methodological training. A total of 134 (62.3%) users believed that WBQs were practical, but 126 (58.6%) had doubts about the reliability of the results. Most of the knowledge and practice topics did not reach a satisfactory level, and some even did not reach a qualified level. A total of 95 (44.2%)-136 (63.3%) of the users had reported difficulties in conducting WBQ survey research, and different participants could have different difficulties according to their characteristics. In addition, 191 (88.8%) users believed training was necessary.

**Conclusions:**

We found that Chinese health workers seriously underestimated and neglected the importance of the WBQ methodology, which may be an important reason for the reduced reporting quality of WBQ survey research. Medical educators need to strengthen methodological training on WBQs, which may help to improve the quality of WBQ survey research.

## Introduction

Questionnaire surveys are an important method in medical epidemiological research and have been applied to scientific research for approximately 100 years [1]. In recent decades, web-based questionnaire (WBQ) survey methods (using computerized self-administered questionnaires stored on a server and accessed via a browser [2]) have gradually become popular worldwide [3,4]. In 2019, the World Health Organization (WHO) published the first digital health guideline, recommending “digital tracking of patients’/clients’ health status and services via mobile devices” [5], which required reliable data collection through digital methods. Thus, with the advent of the digital health era, WBQs are expected to become one of the most acceptable data collection methods.

Currently, WeChat is becoming the most promising scientific social media platform worldwide, especially in China [6]. It has been reported that the number of internet citizens in China has reached 989 million, with an internet penetration rate of 70.4% [7], and the number of WeChat active users worldwide has reached 1.26 billion, most of whom are in China [8]. More importantly, given the popularity of the internet and WeChat and the compatibility between WeChat and WBQ software, the potential difference in population coverage between paper-based questionnaires and WBQs is gradually narrowing [9], which effectively reduces the risk of selection bias of WBQ survey research. Therefore, the platform and audience of WBQ surveys in China have reached an unprecedentedly high level, implying the emergence of a large number of WBQ survey research in the future.

The WBQ has many advantages that traditional questionnaires do not have, but its disadvantages are also obvious (such as selection bias, information bias, problems of data reliability, and repeatability) [2,3,10,11]. Therefore, acceptable WBQ survey research must have a standardized research design, implementation, and reporting to ensure the reliability of the results [12]. Unfortunately, only a few of the existing books and guidelines on questionnaire survey methodology involve a WBQ [2], which may not be compatible with the experience of traditional questionnaire surveys. Bennet et al [13] and Turk et al [14] showed disappointing results in the quality of reporting the studies (most of the studies were underreported, with some items even reaching 98%) after evaluating published WBQ survey research in 2010 and 2018, respectively, indicating that the current reporting quality is unsatisfactory. However, little is known about the reasons that exactly lead to the underreported situation in WBQ survey research.

It seems that in the digital health era with the wide use of WBQs, researchers are more likely to pay attention to the advantages rather than the disadvantages of WBQs, and few researchers recognize the differences between WBQs and traditional questionnaires, suggesting that the WBQ methodology may have long been neglected. Therefore, it is urgent to investigate the current knowledge, attitudes, and practices (KAP) of WBQ methodology among health workers to further guide and improve this situation. For this reason, we developed a WBQ according to the recommendations and current knowledge of WBQ methodology [3,10,11,15] and conducted a cross-sectional survey on the KAP of WBQ methodology among Chinese health workers for the first time. The novelty and importance of our study is that we tried to clarify what makes the current quality of reporting WBQ survey research uneven and unsatisfactory from the root causes (researchers’ KAP in WBQ methodology) to provide a basis for improvement in the future.

## Methods

### Study Design and Participants

From July 8 to July 10, 2022, we held the 2022 Chongqing’s Academic Annual Conference of Child Health Care (web-based) and delivered a lecture on WBQ methodology at the conference on July 8 (Multimedia Appendix 1) [16]. The participants of the conference were mainly health workers of child health care and pediatrics in institutions at all levels. Notably, in China, child health care workers mainly conduct epidemiological research and are one of the most experienced groups in the practice of traditional questionnaires and WBQ surveys [17]. Thus, we chose these participants as the target population to understand the KAP of WBQ among Chinese health workers.

**Figure 1:**
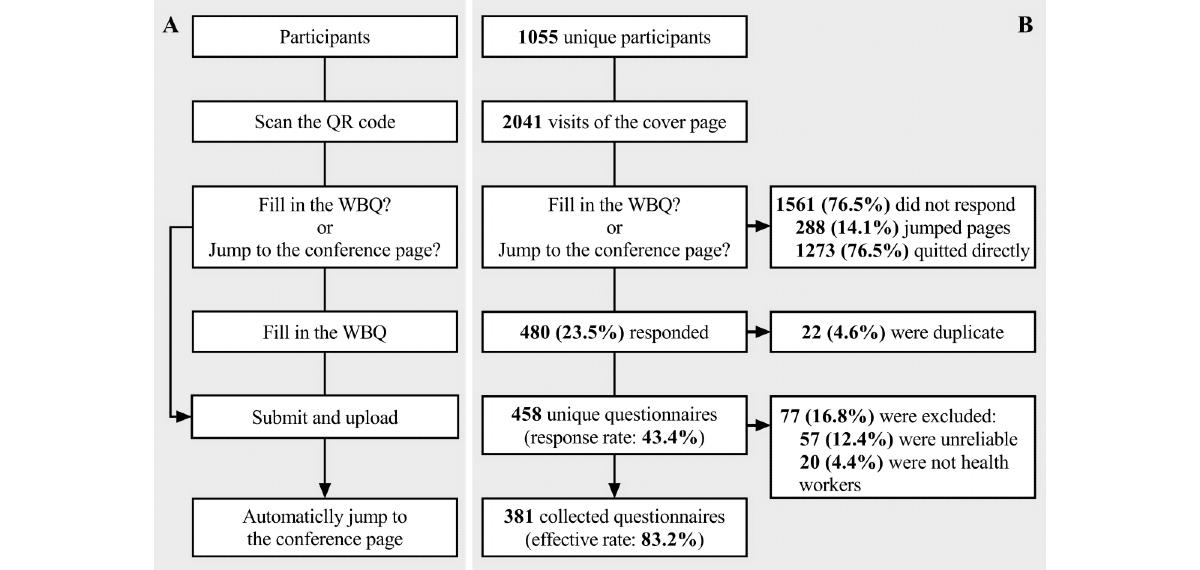
Study design and participants of this study. (A) The process of login for the conference and access to the questionnaire. (B) Flow chart of the questionnaire survey and data processing. QR: quick response; WBQ: web-based questionnaire.

This study was set as the login part of the conference and investigated the participants through a self-developed WBQ from June 7 to July 6, which was before the conference. All participants were recruited without selection and were allowed to decide whether to complete the questionnaire or skip and jump to the conference page directly (Figure 1). This study was an exploratory study, and the main outcome was the KAP of WBQ among Chinese health workers, while the characteristics of the participants who had difficulties in conducting WBQ survey research were the secondary outcomes.

We referred to the ethical issues and suggestions of using social media for recruitment proposed by Gelinas et al [18] and followed the Checklist for Reporting Results of Internet E-Surveys (CHERRIES; Multimedia Appendix 2) and Strengthening the Reporting of Observational Studies in Epidemiology (STROBE; Multimedia Appendix 3) checklists to report the results [15,19].

### Ethics Approval

Our study was reviewed and approved by the Ethics Committee of the Children's Hospital of Chongqing Medical University (No. 2022-216).

### Development of the Questionnaire

We developed a structured WBQ through “Lediaocha.com” (an open web-based questionnaire platform developed by Shanghai Feiguan Information Technology Co, Ltd) and displayed the description of the survey on the cover page (Multimedia Appendix 4). The questionnaire consisted of 48 required questions on 4 pages (14 questions related to general characteristics, 8 questions related to attitude, 11 questions related to knowledge, and 15 questions related to practices). The questions were based on the CHERRIES checklist [15], the recommendations (from Minto et al [3], Ball et al [10], and Regmi et al [11]), and our current knowledge.

Logical display rules were set between relevant questions, which were displayed only after specific conditions were met to avoid false positive answers and save time. We added spaces next to the core vocabulary of each topic to shorten the reading time according to psychological theory (appropriate word spacing is beneficial to Chinese reading) [20], and the whole questionnaire took approximately 5 minutes to complete.

In addition, we set 3 logical progressive questions (“Have you heard of the WBQ?” “Have you conducted the WBQ survey?” “Have you participated in training on WBQs?”), 1 repeated question about age at the beginning and end, and 1 self-evaluation question about the response quality (“The answer is completed and cannot be modified after submission; please help us evaluate the quality of your answer”) to evaluate the reliability of the responses [21].

### Settings of the Questionnaire

The questionnaire was completed anonymously and voluntarily and was accessed and answered through the browser provided with WeChat on a mobile phone or computer. The participants would automatically jump to the conference page after submitting the questionnaire and were allowed to choose to skip the questionnaire directly on the cover page (Figure 1A). The number of answers per account was not limited, but the WeChat nickname and device ID were recorded on the first visit for a duplicate response check. The questionnaire was open access without passwords, which allowed WeChat users to share and promote it. However, the questionnaire could not be retrieved directly by the search engine and was not provided to the public template library.

The serial numbers of questions and completion indicators were displayed while answering, submitted responses could not be reviewed or changed, and unsubmitted responses were not automatically saved. The data stored in the cloud server of “Lediaocha.com” were downloaded and saved by the researchers and then deleted after the survey to avoid potential data security issues. According to the responses, we gave a lecture on WBQ methodology at the conference after the survey, which was used as a response inducement measure.

### Pretest and Promotion

A pretest was conducted before the formal survey. A total of 10 health workers came from the research team, and other institutes were recruited to test the expression of the questions and the process of the survey. We promoted the questionnaire for 30 days before the opening of the conference. Because the hot topics of online public opinion usually have the characteristics of rapid growth and rapid decline [22], we conducted 4 centralized promotions (including WeChat Groups, Moments, and official accounts). The interval between each promotion was no more than 1 week.

### Sample Size Estimation

Currently, there is no clear sample size calculation method for KAP studies. We referred to the sample size estimation method for the quality-of-life study (the sample size is recommended to be 5-10 times the number of items concerned) [23], as the KAP study is a multi–end point questionnaire study and is similar to the quality-of-life study. Thus, the minimum number of valid questionnaires was 170 (5 times of the 34 questions about KAP) to analyze the KAP. In addition, since the number of health workers in China and the proportion of their use of WBQ are unknown, we assumed that the proportion was 50% (which provided the largest calculated sample size compared to other proportions) [24] with an allowable error of 0.05 and a confidence level of 95%. The calculation identified 402 as the minimum sample size through PASS 11.0, and 201 health workers who had used WBQs were expected to be recruited.

### Data Processing

The collected data were directly exported as an Excel (Microsoft Inc) file and checked for inclusion criteria, including health workers, nonduplicate responses, and reliable responses. Duplicate responses were screened by WeChat nickname and device ID and then verified by gender and age. Furthermore, we analyzed the 22 duplicate responses to decide which one should be excluded and found that only 4 duplicate responses were not easy to judge (Multimedia Appendix 5). Thus, we excluded the 4 later duplicate responses according to psychological theory, which indicates that the first impression is more accurate [25], as the small proportion (4/458, 0.9%) was unlikely to affect the results. A data reliability check was conducted through the 3 groups of preset questions, and the response was determined to be unreliable and excluded when any of the preset questions did not meet the requirements. Finally, the responses of nonhealth workers were excluded.

### Statistical Analysis

Data analysis was performed with GraphPad Prism 9. Qualitative data were described as frequencies (percentages), and quantitative data were described as medians (interquartile range, IQR) after normality testing. In this study, 50% and 75% were defined as “qualified” and “satisfactory,” respectively, for the proportion of knowledge and practice questions. The chi-square test and Mann-Whitney *U* test were used to analyze the general characteristics (including sex, age, occupation, specialized subject, positional title, and hospital level) between the users and nonusers of WBQ. In addition, multivariate logistic regression was used to analyze the general characteristics of the users who had difficulties in WBQ survey research. Further subgroup analysis was not conducted, as the sample size would be insufficient. *P*<.05 was considered statistically significant.

## Results

### General Characteristics of the Participants

**Figure 2:**
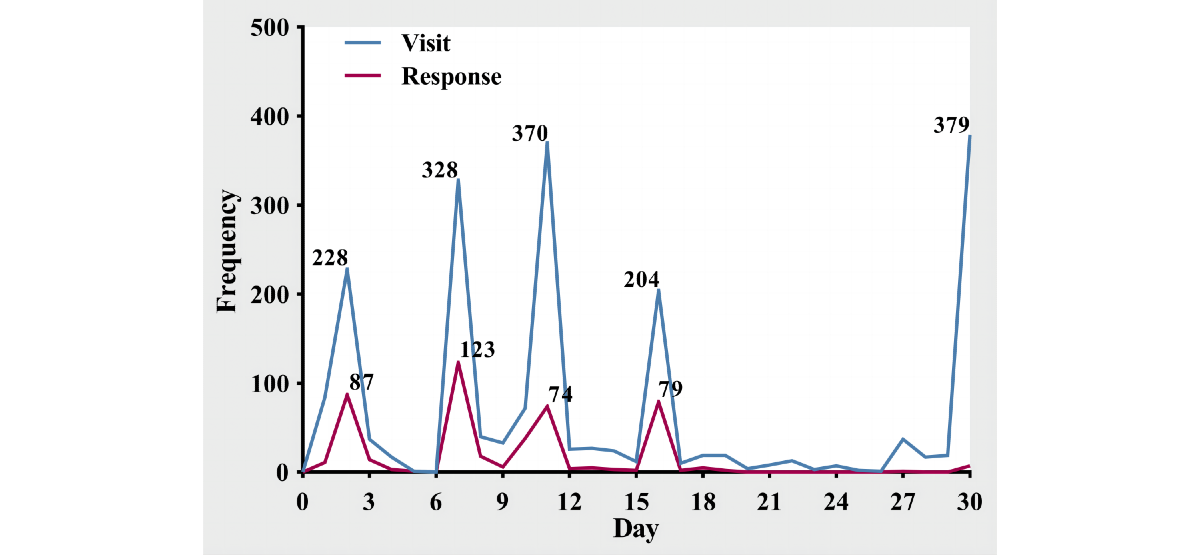
The visits and responses to the web-based questionnaire.

Figure 2 shows the visits and responses to the questionnaire. The number of recruits increased and then decreased rapidly in a pulse manner after each promotion. The questionnaire was visited 2041 times by 1055 participants (the duplicate visit rate was 48.3%) and was responded to 480 times by 458 participants (the duplicate response rate was 4.6% and the unique response rate was 43.4%). A total of 381 valid questionnaires were finally included in the analysis (the effective rate was 83.2%), and the response time was 3.7 (IQR 2.6-5.1) minutes (Figure 1B).

Table 1 shows the general characteristics of the participants. Women accounted for 90.6% (345/381), most respondents were 37 (IQR 11) years old, and doctors accounted for 70.1% (267/381). A total of 91.6% (349/381) of the participants had heard of WBQs, 88.3% (308/349) believed that WBQs were more suitable for investigation, and 51.9% (181/349) believed that they were easy to use. A total of 56.4% (215/381) of the participants had used WBQs for investigation (users). Among them, 11.6% (25/215) had used a WBQ once, 35.3% (76/215) had used it 2-3 times, and 53.0% (114/215) had used it more than 4 times. However, only 44.2% (95/215) had ever received training. Further analyses were based on the 215 users of WBQ, and the general characteristics (including sex, age, occupation, specialized subject, positional title, and hospital level) between the users and nonusers were not significantly different (*P*>.05).

**Table 1:** General characteristics of the participants (N=381)

Characteristics			Statistical results
**Sex, n (%)**			
	Female	345 (90.6)	
	Male	36 (9.4)	
Age (years), median (IQR)			37 (11)
**Occupation, n (%)**			
	Doctor	267 (70.1)	
	Nurse	67 (17.6)	
	Others	47 (12.3)	
**Specialized subject, n (%)**			
	Child health care	242 (63.5)	
	Pediatrics	81 (21.3)	
	Others	58 (15.2)	
**Positional title, n (%)**			
	None or junior	157 (41.2)	
	Intermediate	150 (39.4)	
	Senior	74 (19.4)	
**Hospital level, n (%)**			
	None or grade 1	88 (32.1)	
	Grade 2	153 (40.2)	
	Grade 3	140 (36.7)	
Had heard of WBQs^a^, n (%)			349 (91.6)
Had used WBQs for investigation, n (%)			215 (56.4)
Had received a WBQ training, n (%)			103 (27.0)

^a^WBQ: web-based questionnaire.

### Knowledge and Practices of WBQ Methodology

**Figure 3:**
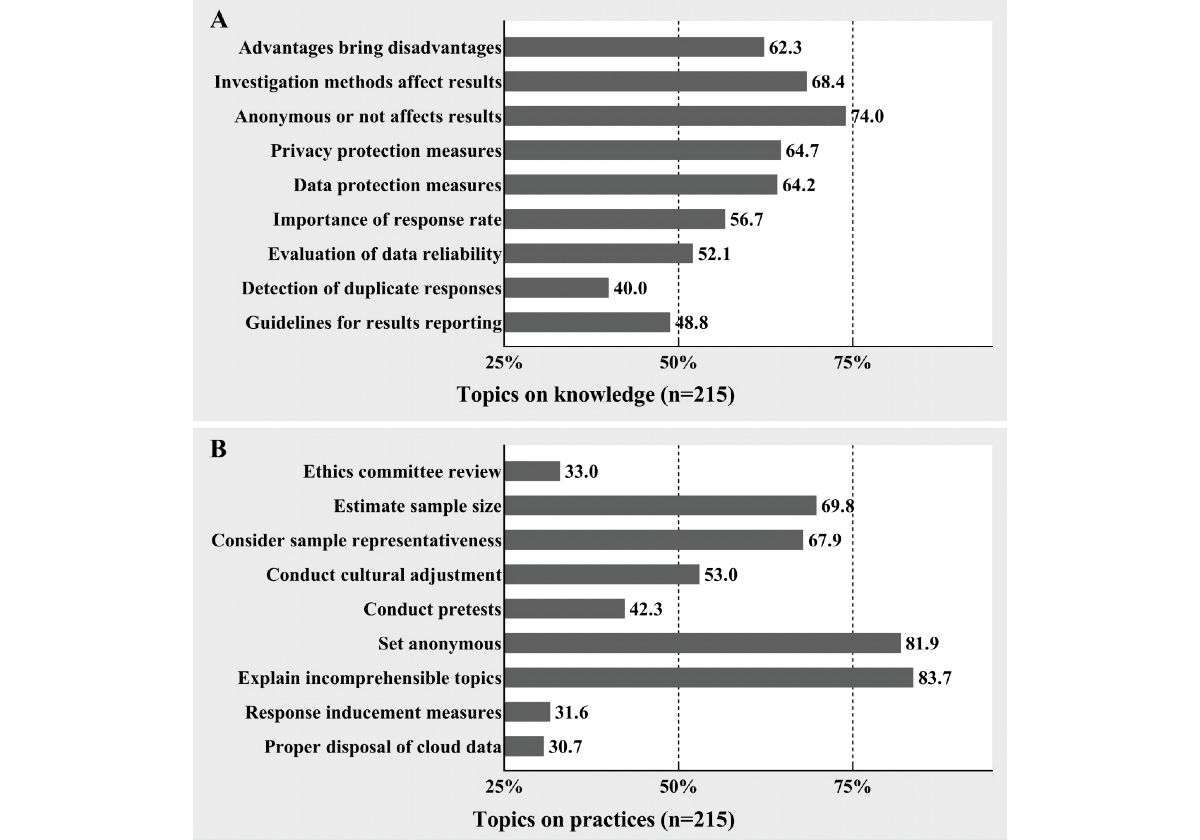
Knowledge and practices of web-based questionnaire methodology. (A) The proportion of knowledge topics. (B) The proportion of practice topics.

Figure 3 shows the knowledge and practices of the methodology among the users (N=215). In the knowledge topics, “detection of duplicate responses” (86, 40.0%) and “guidelines for results reporting” (105, 48.8%) did not reach a qualified level, and no topics reached a satisfactory level. In the practice topics, “ethics committee review” (71, 33.0%), “conduct pretests” (91, 42.3%), “response inducement measures” (68, 31.6%), and “proper disposal of cloud data” (66, 30.7%) did not reach a qualified level. Only “set anonymous” (176, 81.9%) and “explain incomprehensible topics” (180, 83.7%) reached a satisfactory level.

### Attitude on WBQs

Table 2 shows the attitude toward conducting web-based surveys among the users (N=215). Among them, 72.6% (156) believed that a WBQ was easy to accept, and 62.3% (134) believed that a WBQ was practical, but 58.6% (126) had doubts about the reliability of the results. In addition, 88.8% (191) of the users believed that training was necessary. Regarding the difficulties of WBQ surveys, “quality control” (136, 63.3%), “study and questionnaire design” (134, 62.3%), “data processing and statistical analysis” (128, 59.5%), and “response rate of participants” (126, 58.6%) were the most difficult aspects.

The multivariate analyses (Table 3) showed that users who were male (odds ratio [OR] 0.35, 95% CI 0.14-0.87, *P*=.03) and younger (OR 0.95, 95% CI 0.90-0.99, *P*=.03) were less likely to report difficulties in response issues. Doctors were more likely to report difficulties in quality issues than nurses (OR 0.34, 95% CI 0.15-0.76, *P*=.009) and other health workers (OR 0.32, 95% CI 0.11-0.97, *P*=.04). Pediatric workers (OR 2.54, 95% CI 1.15-5.61, *P*=.02) were more likely to report difficulties in design issues than child health care workers. Users with senior positional titles were less likely to report difficulties in promotion issues (OR 0.26, 95% CI 0.08-0.87, *P*=.03) but were more likely to report difficulties in response issues (OR 3.26, 95% CI 1.01-10.60, *P*=.049) than users with junior/none titles.

**Table 2:** Attitude on web-based questionnaires (N=215)

Topics			Respondents, n (%)
**Respondents’ acceptance of WBQs^a^**			
	No feeling	35 (16.3)	
	Most people refuse	24 (11.2)	
	Most people accept	156 (72.6)	
**The practicability of WBQs**			
	Completely impractical	1 (0.5)	
	Slightly or moderately practical	80 (37.2)	
	Very or extremely practical	134 (62.3)	
**Reliability of WBQs**			
	Completely unreliable	4 (1.9)	
	Slightly or moderately reliable	122 (56.7)	
	Very or extremely reliable	89 (41.4)	
**Training of WBQ survey study**			
	Does not matter or not necessary	24 (11.2)	
	Necessary	191 (88.8)	
**Difficulties in WBQ survey study**			
	Study and questionnaire design	134 (62.3)	
	Advertising and publicity	95 (44.2)	
	Response rate of participants	126 (58.6)	
	Quality control	136 (63.3)	
	Data processing and statistical analysis	128 (59.5)	
	Report of results	98 (45.6)	

^a^WBQ: web-based questionnaire.

**Table 3:** Multivariate analyses of the characteristics in users who had difficulties in web-based questionnaire survey research (n=215).

Characteristics			Design, OR^a^ (95% CI)		Promotion, OR (95% CI)		Response, OR (95% CI)		Quality, OR (95% CI)		Analysis, OR (95% CI)		Report, OR (95% CI)
**Sex**													
	Female	Reference		Reference		Reference		Reference		Reference		Reference	
	Male	0.98 (0.38-2.52)		1.09 (0.43-2.73)		0.35^b^ (0.14-0.87)		0.47 (0.18-1.18)		0.41 (0.16-1.04)		0.96 (0.38-2.40)	
Age (years)			1.03 (0.98-1.08)		0.99 (0.95-1.04)		0.95^b^ (0.90-0.99)		0.96 (0.92-1.01)		0.99 (0.94-1.04)		1.01 (0.96-1.06)
**Occupation**													
	Doctor	Reference		Reference		Reference		Reference		Reference		Reference	
	Nurse	1.26 (0.57-2.77)		0.82 (0.37-1.79)		0.92 (0.42-2.01)		0.34^c^ (0.15-0.76)		0.57 (0.26-1.25)		1.06 (0.49-2.27)	
	Others	3.20 (0.99-10.34)		0.59 (0.20-1.72)		1.14 (0.39-3.37)		0.32^b^ (0.11-0.97))		0.62 (0.21-1.86)		0.55 (0.18-1.68)	
**Specialized subject**													
	Child health care	Reference		Reference		Reference		Reference		Reference		Reference	
	Pediatrics	2.54^b^ (1.15-5.61)		1.02 (0.50-2.11)		1.28 (0.62-2.68)		1.97 (0.86-4.49)		0.77 (0.37-1.58)		0.83 (0.41-1.68)	
	Others	0.64 (0.25-1.59)		0.63 (0.25-1.58)		1.16 (0.47-2.91)		1.37 (0.53-3.52)		0.49 (0.20-1.21)		0.50 (0.20-1.27)	
**Positional titles**													
	Junior/none	Reference		Reference		Reference		Reference		Reference		Reference	
	Intermediate	1.03 (0.48-2.22)		0.64 (0.30-1.34)		1.78 (0.83-3.81)		0.91 (0.41-2.04)		0.78 (0.36-1.70)		1.05 (0.50-2.20)	
	Senior	0.79 (0.25-2.54)		0.26^b^ (0.08-0.87)		3.26^b^ (1.01-10.60)		1.18 (0.35-4.00)		0.67 (0.21-1.34)		0.97 (0.32-2.97)	
**Hospital level**													
	Grade 1/none	Reference		Reference		Reference		Reference		Reference		Reference	
	Grade 2	1.04 (0.47-2.31)		1.17 (0.53-2.59)		1.06 (0.47-2.37)		0.61 (0.27-1.39)		0.65 (0.29-1.46)		0.85 (0.39-1.86)	
	Grade 3	1.07 (0.46-2.50)		0.99 (0.42-2.32)		0.90 (0.38-2.10)		1.40 (0.57-3.41)		0.94 (0.40-2.23)		0.87 (0.38-2.01)	

^a^OR: odds ratio.

^b^*P*<.05.

^c^*P*<.01.

## Discussion

### Principal Findings

In 2010, van Gelder et al [26] proposed that WBQs could be expected to become the future of epidemiology, but the design of the questionnaire would have a significant impact on the results. Since then, Bennet et al [13] and Turk et al [14] have reviewed the published WBQ survey research and found that the quality of reporting the research was not optimal. To clarify the possible reasons for the unsatisfactory reporting quality of WBQ survey research, we described the KAP of the WBQ methodology among Chinese health workers for the first time. Our study found that the participants who had heard of WBQs generally believed it was suitable for investigation (308/349, 88.3%), and more than half (181/349, 51.9%) believed it was easy to use. However, further analysis of the users showed that most of the knowledge and practice topics did not reach a satisfactory level, and some even did not reach a qualified level. Therefore, although WBQs are regarded as the most promising epidemiological research method, the methodology is seriously underestimated and neglected, which may explain why the research is underreported.

### The Differences Between WBQs and Traditional Questionnaires

It is known that the WBQ methodology directly affects the reliability of the results [10]. Our study showed that 62.3% (134/215) of the users believed that it was very practical, but more than half of the users (126/215, 58.6%) also had doubts about the reliability of the results. The possible reason is that WBQ users do not fully understand the influences that WBQs bring to the results, causing a lack of consideration and confidence in the reliability. More importantly, our study showed that more than half of the users (110/215, 51.2%) did not even know the guidelines for reporting WBQ survey research, indicating that they do not treat WBQ as a survey method different from traditional questionnaires. However, the current research shows that there are many differences between WBQ and traditional questionnaires. Thus, researchers need to know the potential influences when applying WBQ to research.

The most concerning topic is that which is more accurate in the results of WBQs and traditional questionnaires. Although some studies have shown that WBQs and traditional questionnaires might obtain different results when dealing with subjective or sensitive topics [21,27,28], many studies have shown that WBQs have the same internal consistency as paper questionnaires and have even higher reliability when dealing with private topics [29-33]. In fact, the reasons why different survey methods produce different results are partly related to the complexity and variability of human psychology. For some topics related to privacy or social stigmatization, face-to-face surveys may be more vulnerable to social expectation bias than WBQs [34-36], but some studies have shown that WBQs have no advantage in this regard [37]. In addition, for some topics that are difficult to understand, WBQ may be more prone to information bias than face-to-face surveys. A small number of WBQ participants (5/515, 1%) reported comprehension problems even after careful cultural adjustment and pretesting, as previously described [21]. Our study showed that “set anonymous” (176/215, 81.9%) and “explain difficult topics” (180/215, 83.7%) reached a satisfactory level, indicating that the researchers had fully recognized these two aspects. However, anonymity also brings its problems, that is, it is difficult to verify when the data are difficult to explain.

The other topic of concern could be that different survey methods could cover different populations, that is, researchers should consider the sample size and representativeness of the sample. In this regard, our study showed that “estimate sample size” (150/215, 69.8%) and “consider sample representativeness” (146/215, 67.9%) had reached a qualified level. In fact, studies have shown that some groups of people are not able to easily access WBQs (such as older adult people) [38-40]. However, in recent years, an increasing number of older adult people have begun to use WeChat and carry out communications about health issues [41]. At the same time, the coverage of WBQs has been further expanded, so the low response rate does not necessarily increase selection bias [9]. However, it is not enough to rely only on previous research results to determine the selection bias of WBQ. Researchers should consider creating advertisements targeting underrepresented populations [40] or adjusting publicity strategies [42].

### Neglect of the WBQ Methodology

To standardize the research using WBQs, Eysenbach et al [15] formulated a checklist (CHERRIES) of the results reported in 2004, which provided several recommendations to improve the quality of WBQ survey research and are still highly applicable. In the past decade, Mario et al [2], Minto et al [3], Ball et al [10], and Regmi et al [11] all proposed recommendations on the design and implementation of WBQ surveys. However, given the unsatisfactory reporting quality of WBQ survey research according to the literature reviews [13,14], these recommendations seemed to have little effect on improving the reporting quality. Our study further showed that the current KAP of the WBQ methodology among Chinese health workers was not ideal, as most of the items were far from satisfactory. Although our study focused on Chinese health workers, it provided evidence on the lack of researchers’ awareness of the WBQ methodology, which may explain the insufficient effects of current recommendations.

It is worth mentioning that with the development of internet companies, an increasing amount of research has been conducted through open web-based survey platforms, which may bring additional problems for the WBQ methodology. The most prominent problems involve privacy protection and data security and have gradually become the focus of ethics committees [1]. Our study showed that only 30.7% (66/215) of the WBQ users would dispose of cloud data after the survey, and 33.0% (71/215) would submit it to the ethics committee for review, which could increase the potential risk of data disclosure. Although the related items in the CHERRIES checklist are currently insufficient [15], we recommend that researchers properly deal with cloud data after the survey (such as saving it by a specially assigned researcher after downloading).

Another problem could be the strategy of recruiting through social media, which is related to the response rate that was reported with difficulties by 58.6% (126/215) of the users. In 2005, Dannetun et al [43] showed that most of the responses (70%) to WBQs were received within 7 days after promotion. However, our study showed that when the questionnaire was promoted through social media, each promotion was only valid for 1-2 days, and the recruitment potential was basically lost after the valid period. This may be related to the fact that hot topics of web-based public opinion have their own life cycle [22], the topics of our survey were not attractive enough, and the response inducement measures were not immediate. However, it may also be related to the current popular fast-food culture. Therefore, although WBQs are easy to promote, researchers may not obtain the expected sample size if they do not know these characteristics when formulating promotion strategies, which may lead to excessive promotions. However, it remains unclear whether excessive promotion will have a negative impact on the sample.

### Requirement for Methodological Training of WBQs

The current KAP of the WBQ methodology is not ideal, suggesting that education or training is obviously insufficient. The surveys of Edirippulige et al [44] and Machleid et al [45] showed that medical students had a great demand for digital health education and training and were eager to improve their knowledge of digital health. Our study also showed that less than half (95/215, 44.2%) of the users had received training before conducting WBQ surveys, while most of them (191/215, 88.8%) believed that training was necessary. Considering the frequency with which the users used WBQs in our study (190/215, 88.4% had used them more than 2 times and were considered well experienced), their significant demand for methodological training is credible.

For the education or training of WBQ methodology, our study provided content (such as the unqualified topics) that should be strengthened. In addition, “quality control” (136/215, 63.3%), “study and questionnaire design” (134/215, 62.3%), “data processing and statistical analysis” (128/215, 59.5%), and “response rate of participants” (126/215, 58.6%) were the most difficult aspects according to the users’ self-reports. Different users had different difficulties in WBQ surveys. For example, participants with senior positional titles reported fewer difficulties in promotion issues but more difficulties in response issues, which might be related to the resources, experiences, and skills that users had. Therefore, future medical educators should also consider targeted content while increasing the number of methodological trainings on WBQs.

### Strengths and Limitations

The strengths and limitations of this study require careful consideration. First, this study focused on the situation of unsatisfactory reporting quality of WBQ survey research, which is an international problem and provided unique insights on improving the quality of WBQ survey research from the perspective of researchers. Second, this study was designed and conducted by a well-experienced WBQ survey team, which guaranteed the reliability of the results. Third, this study discussed the current WBQ survey methods, which are meaningful not only for medical educators but also for WBQ users.

Despite its strengths, this study has some limitations. First, for selection bias, this study is focused on Chinese health workers, so that the results could not be simply extended to other populations but still provided clues. This study took an academic conference as the context, which may make the participants more experienced in WBQs than the average level and cause overestimation of the real KAP of Chinese health workers. In addition, this study did not analyze the potential differences between participants and nonparticipants due to lack of data on the nonresponders. However, since our outcomes were based on WBQ users, we analyzed the differences between users and nonusers and found no significant difference. Second, for response rate, although the response rate of our study (458/1055, 43.4%) was lower than the mean level (51.3%) of public health research using WBQ from 2010 to 2015 [46], it could be underestimated because many nonparticipants in social media could also access the questionnaire. Third, for network technology, this study was based on an open WBQ platform, which improved the convenience but also increased the lack of specific data (such as login files) [47], leading to the unknown characteristics of nonresponders.

### Conclusions

In summary, this study found that Chinese health workers seriously underestimated and neglected the importance of the WBQ methodology, which may be an important reason for the reduced reporting quality of WBQ survey research. The accessibility and convenience of WBQs should not be the reason researchers neglect their methodology. Medical educators need to strengthen methodological training on WBQs, which may help to improve the reliability of a large number of WBQ survey studies in the digital health era.

## Appendix

Multimedia Appendix 1 The electronic poster of the conference.

Multimedia Appendix 2 CHERRIES checklist.

Multimedia Appendix 3 STROBE checklist.

Multimedia Appendix 4 Cover page of the WBQ.

Multimedia Appendix 5 Analysis and exclusion of duplicate questionnaires.

## Abbreviations

CHERRIES: Checklist for Reporting Results of Internet E-Surveys
KAP: knowledge, attitude, and practices
WBQ: web-based questionnaire

## References

[1] Shao G, Xie J (2021). The state and problems of online survey in China [in Chinese]. Journal of Hunan University (Social Sciences).

[2] Mario C, Katja LM, Vasja V (2015). Web Survey Methodology.

[3] Minto C, Vriz GB, Martinato M, Gregori D (2017). Electronic Questionnaires Design and Implementation. Open Nurs J.

[4] Marra C, Chen JL, Coravos A, Stern AD (2020). Quantifying the use of connected digital products in clinical research. NPJ Digit Med.

[5] World Health Organization (2019). WHO guideline: recommendations on digital interventions for health system strengthening. Licence: CC BY-NC-SA 3.0 IGO.

[6] Nature Methods (2020). Science on WeChat. Nat Methods.

[7] (2021). The 47th statistical report on China’s internet development. China Internet Network Information Center.

[8] (2022). Most popular global mobile messaging apps 2022. Statista.

[9] Ebert JF, Huibers L, Christensen B, Christensen MB (2018). Paper- or web-based questionnaire invitations as a method for data collection: cross-sectional comparative study of differences in response rate, completeness of data, and financial cost. J Med Internet Res.

[10] Ball HL (2019). Conducting Online Surveys. J Hum Lact.

[11] Regmi PR, Waithaka E, Paudyal A, Simkhada P, van Teijlingen E (2016). Guide to the design and application of online questionnaire surveys. Nepal J Epidemiol.

[12] Krantz JH, Reips UD (2017). The state of web-based research: a survey and call for inclusion in curricula. Behav Res Methods.

[13] Bennett C, Khangura S, Brehaut JC, Graham ID, Moher D, Potter BK, Grimshaw JM (2010). Reporting guidelines for survey research: an analysis of published guidance and reporting practices. PLoS Med.

[14] Turk T, Elhady MT, Rashed S, Abdelkhalek M, Nasef SA, Khallaf AM, Mohammed AT, Attia AW, Adhikari P, Amin MA, Hirayama K, Huy NT (2018). Quality of reporting web-based and non-web-based survey studies: what authors, reviewers and consumers should consider. PLoS One.

[15] Eysenbach G (2004). Improving the quality of web surveys: the checklist for reporting results of internet e-surveys (CHERRIES). J Med Internet Res.

[16] Child Health Care Branch of Chongqing Medical Association (2022). Notice of the 2022 Chongqing Medical Association Children's Health Care Branch Academic Annual Conference and Children's Food Allergy Class and Child Health Care Continuing Education Advanced Class. http://www.cqma.cn/news/2281.html.

[17] Li H (2022). The Principle and Practice of Pediatric Primary Care. 2 ed.

[18] Gelinas L, Pierce R, Winkler S, Cohen IG, Lynch HF, Bierer BE (2017). Using social media as a research recruitment tool: ethical issues and recommendations. Am J Bioeth.

[19] von Elm E, Altman DG, Egger M, Pocock SJ, Gøtzsche PC, Vandenbroucke JP, STROBE Initiative (2007). The Strengthening the Reporting of Observational Studies in Epidemiology (STROBE) statement: guidelines for reporting observational studies. Lancet.

[20] Oralova G, Kuperman V (2021). Effects of spacing on sentence reading in Chinese. Front Psychol.

[21] Fang H, Xian R, Ma Z, Lu M, Hu Y (2021). Comparison of the differences between web-based and traditional questionnaire surveys in pediatrics: comparative survey study. J Med Internet Res.

[22] He Y, Li J, Zhu M, Xu D (2018). Life cycle identification and analysis of microblog hot topics.

[23] Yu L, Fang J, Tian L, Jin H (2015). Advanced Medical Statistics. 2nd ed.

[24] Sun Z, Xu Y, Yan Y, Wang T, Liu H, Ma J (2020). Medical Statistics. 5th ed. [in Chinese].

[25] Biesanz JC, Human LJ, Paquin A, Chan M, Parisotto KL, Sarracino J, Gillis RL (2011). Do we know when our impressions of others are valid?: evidence for realistic accuracy awareness in first impressions of personality. Soc Psychol Personal Sci.

[26] van Gelder MM, Bretveld RW, Roeleveld N (2010). Web-based questionnaires: the future in epidemiology?. Am J Epidemiol.

[27] Braekman E, Charafeddine R, Demarest S, Drieskens S, Berete F, Gisle L, Van der Heyden J, Van Hal G (2020). Comparing web-based versus face-to-face and paper-and-pencil questionnaire data collected through two Belgian health surveys. Int J Public Health.

[28] Milton AC, Ellis LA, Davenport TA, Burns JM, Hickie IB (2017). Comparison of self-reported telephone interviewing and web-based survey responses: findings from the second Australian young and well national survey. JMIR Ment Health.

[29] Benedik E, Koroušić Seljak B, Simčič M, Rogelj I, Bratanič B, Ding EL, Orel R, Fidler Mis N (2014). Comparison of paper- and web-based dietary records: a pilot study. Ann Nutr Metab.

[30] Terluin B, Brouwers EPM, Marchand MAG, de Vet HCW (2018). Assessing the equivalence of web-based and paper-and-pencil questionnaires using differential item and test functioning (DIF and DTF) analysis: a case of the four-dimensional symptom questionnaire (4DSQ). Qual Life Res.

[31] Zhang C, Sun Z, Yang J, Xu T, Zhu L, Lang J (2020). Comparative validity and reliability of the WeChat-based electronic and paper-and-pencil versions of the PISQ-12 for collecting participant-reported data in Chinese. Menopause.

[32] Tanaka M, Saito M, Takahashi M, Adachi M, Nakamura K (2021). Interformat reliability of web-based parent-rated questionnaires for assessing neurodevelopmental disorders among preschoolers: cross-sectional community study. JMIR Pediatr Parent.

[33] Sun Z, Zhu L, Liang M, Xu T, Lang J (2016). The usability of a WeChat-based electronic questionnaire for collecting participant-reported data in female pelvic floor disorders: a comparison with the traditional paper-administered format. Menopause.

[34] Lippitt M, Reese Masterson A, Sierra A, Davis AB, White MA (2014). An exploration of social desirability bias in measurement of attitudes toward breastfeeding in public. J Hum Lact.

[35] Crutzen R, Göritz AS (2010). Social desirability and self-reported health risk behaviors in web-based research: three longitudinal studies. BMC Public Health.

[36] King BM (2022). The influence of social desirability on sexual behavior surveys: a review. Arch Sex Behav.

[37] Gnambs T, Kaspar K (2017). Socially desirable responding in web-based questionnaires: a meta-analytic review of the candor hypothesis. Assessment.

[38] Graefe A, Mowen A, Covelli E, Trauntvein N (2011). Recreation participation and conservation attitudes: differences between mail and online respondents in a mixed-mode survey. Hum Dimens Wildl.

[39] Cantuaria ML, Blanes-Vidal V (2019). Self-reported data in environmental health studies: mail vs. web-based surveys. BMC Med Res Methodol.

[40] Farr DE, Battle DA, Hall MB (2022). Using facebook advertisements for women's health research: methodology and outcomes of an observational study. JMIR Form Res.

[41] Zhang X, Xu X, Cheng J (2021). WeChatting for health: what motivates older adult engagement with health information. Healthcare (Basel).

[42] Zhang Y, Xia T, Huang L, Yin M, Sun M, Huang J, Ni Y, Ni J (2019). Factors influencing user engagement of health information disseminated by Chinese provincial centers for disease control and prevention on WeChat: observational study. JMIR Mhealth Uhealth.

[43] Dannetun E, Tegnell A, Giesecke J (2007). Parent's attitudes towards hepatitis B vaccination for their children: a survey comparing paper and web questionnaires, Sweden 2005. BMC Public Health.

[44] Edirippulige S, Gong S, Hathurusinghe M, Jhetam S, Kirk J, Lao H, Leikvold A, Ruelcke J, Yau NC, Zhang Q, Armfield N, Senanayake B, Zhou X, Smith AC, Judd M, Coulthard MG (2022). Medical students' perceptions and expectations regarding digital health education and training: A qualitative study. J Telemed Telecare.

[45] Machleid F, Kaczmarczyk R, Johann D, Balčiūnas J, Atienza-Carbonell B, von Maltzahn F, Mosch L (2020). Perceptions of digital health education among European medical students: mixed methods survey. J Med Internet Res.

[46] Blumenberg C, Barros AJD (2018). Response rate differences between web and alternative data collection methods for public health research: a systematic review of the literature. Int J Public Health.

[47] Vu KPL, Proctor RW, Michael M, Ulf-Dietrich R (2011). Behavioral research and data collection via the internet. Handbook of Human Factors in Web Design.

