# Supplementary figures and images for: The Current Knowledge, Attitudes, and Practices of the Neglected Methodology of Web-Based Questionnaires Among Chinese Health Workers: Web-Based Questionnaire Study
Source: J Med Internet Res. 2023 Jan 27;25:e41591. doi: 10.2196/41591 (PMC9919466; doi:10.2196/41591)

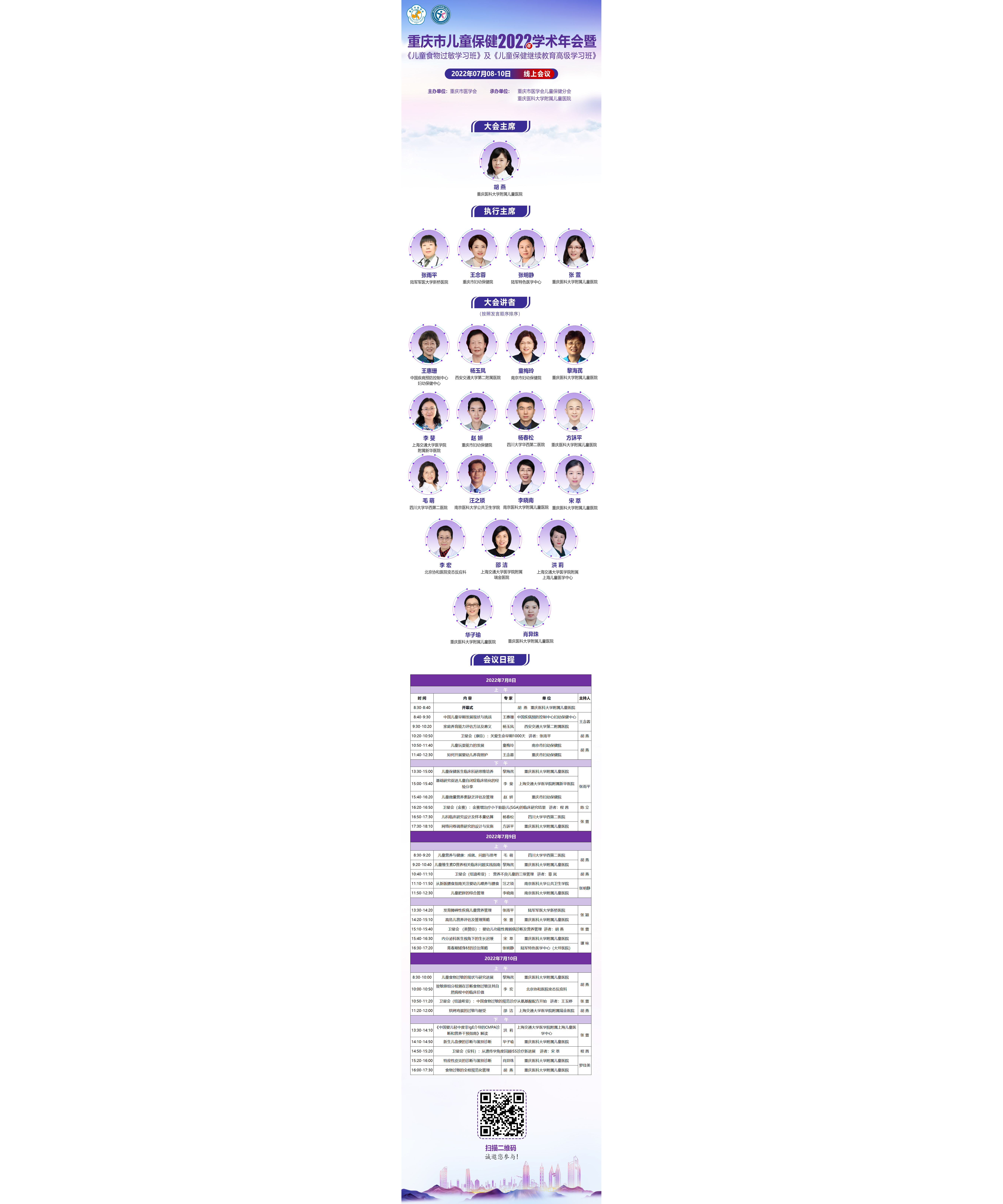

Supplement: Multimedia Appendix 1 [file jmir_v25i1e41591_app1.png]

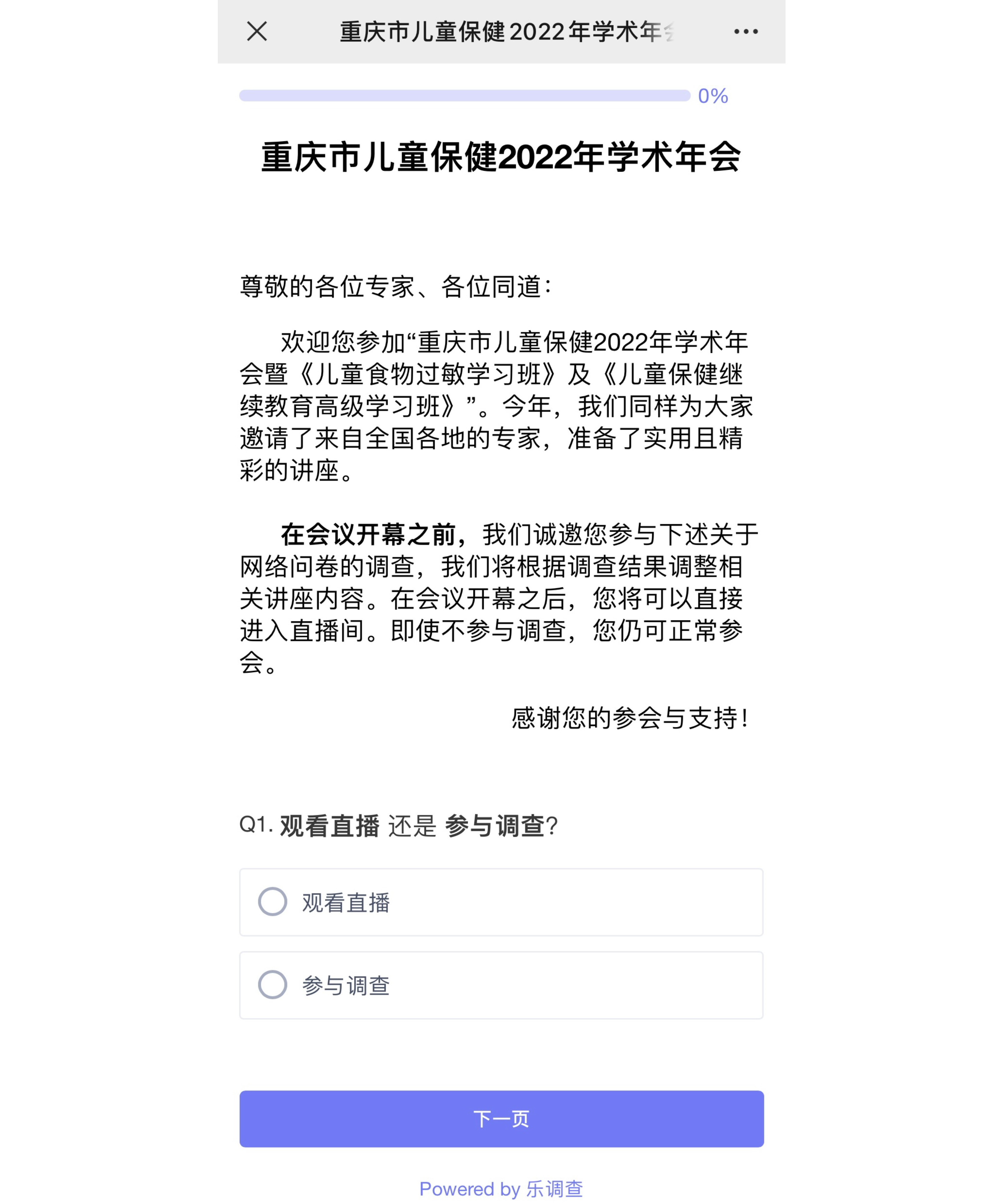

Supplement: Multimedia Appendix 4 [file jmir_v25i1e41591_app4.png]
