# Supplementary material for: The Current Knowledge, Attitudes, and Practices of the Neglected Methodology of Web-Based Questionnaires Among Chinese Health Workers: Web-Based Questionnaire Study
Source: J Med Internet Res. 2023 Jan 27;25:e41591. doi: 10.2196/41591 (PMC9919466; doi:10.2196/41591)
Supplement: Multimedia Appendix 5 [file jmir_v25i1e41591_app5.pdf]

The neglected methodology of web-based questionnaires: the current knowledge, attitudes, and practices among Chinese health workers

Appendix 5. Analysis and exclusion of duplicate questionnaires

Participants	Decision	Reasons for exclusion
**A**		
1	Included	
2	Excluded	It was judged to be unreliable because all questions about the KAP were negative answers.
3	Excluded	It was judged to be not unreliable according to the repeated questions.
**B**		
1	Included	
2	Excluded	It was judged to be unreliable according to the participant's self-report.
3	Excluded	It was judged to be unreliable according to the participant's self-report.
**C**		
1	Included	
2	Excluded	It could not be judged but was excluded according to the first impression theory.
**D**		
1	Included	
2	Excluded	It was identical to the first questionnaire.
**E**		
1	Excluded	It was judged to be unreliable because all questions were negative answers.
2	Included	
3	Excluded	It was identical to the second questionnaire.
**F**		
1	Included	
2	Excluded	It was identical to the first questionnaire.
3	Excluded	It was judged to be unreliable because it was different from the first two questionnaires.
**G**		
1	Included	
2	Excluded	It was judged to be unreliable according to the participant's self-report.
**H**		
1	Included	
2	Excluded	It was identical to the first questionnaire.

**I**			
	1	Included	
	2	Excluded	It was judged to be unreliable according to the participant's self-report.
**J**			
	1	Included	
	2	Excluded	It was judged to be unreliable because all questions were negative answers.
**K**			
	1	Included	
	2	Excluded	It was identical to the first questionnaire.
**L**			
	1	Included	
	2	Excluded	It could not be judged but was excluded according to the first impression theory.
**M**			
	1	Excluded	It was judged to be unreliable because all questions were negative answers.
	2	Included	
**N**			
	1	Included	
	2	Excluded	It could not be judged but was excluded according to the first impression theory.
**O**			
	1	Included	
	2	Excluded	It could not be judged but was excluded according to the first impression theory.
**P**			
	1	Included	
	2	Excluded	It was judged to be unreliable according to the participant's self-report.
**Q**			
	1	Included	
	2	Excluded	It was judged to be unreliable because all questions were negative answers.
**R**			
	1	Included	
	2	Excluded	It was judged to be unreliable according to the participant's self-report.
